# The Laboratory Diagnosis and Follow Up of Strongyloidiasis: A Systematic Review

**DOI:** 10.1371/journal.pntd.0002002

**Published:** 2013-01-17

**Authors:** Ana Requena-Méndez, Peter Chiodini, Zeno Bisoffi, Dora Buonfrate, Eduardo Gotuzzo, José Muñoz

**Affiliations:** 1 Barcelona Centre for International Health Research (CRESIB, Hospital Clínic-Universitat de Barcelona), Barcelona, Spain; 2 Hospital for Tropical Diseases, London School of Hygiene and Tropical Medicine, London, United Kingdom; 3 Centre for Tropical Diseases (CTD), Sacro Cuore Hospital, Verona, Italy; 4 Instituto de Medicina Tropical Alexander von Humboldt, Universidad Peruana Cayetano Heredia, Lima, Peru; George Washington University, United States of America

## Abstract

**Background:**

Strongyloidiasis is frequently under diagnosed since many infections remain asymptomatic and conventional diagnostic tests based on parasitological examination are not sufficiently sensitive. Serology is useful but is still only available in reference laboratories. The need for improved diagnostic tests in terms of sensitivity and specificity is clear, particularly in immunocompromised patients or candidates to immunosuppressive treatments. This review aims to evaluate both conventional and novel techniques for the diagnosis of strongyloidiasis as well as available cure markers for this parasitic infection.

**Methodology/Principal Findings:**

The search strategy was based on the data-base sources MEDLINE, Cochrane Library Register for systematic review, EmBase, Global Health and LILACS and was limited in the search string to articles published from 1960 to August 2012 and to English, Spanish, French, Portuguese and German languages. Case reports, case series and animal studies were excluded. 2003 potentially relevant citations were selected for retrieval, of which 1649 were selected for review of the abstract. 143 were eligible for final inclusion.

**Conclusions:**

Sensitivity of microscopic-based techniques is not good enough, particularly in chronic infections. Furthermore, techniques such as Baermann or agar plate culture are cumbersome and time-consuming and several specimens should be collected on different days to improve the detection rate. Serology is a useful tool but it might overestimate the prevalence of disease due to cross-reactivity with other nematode infections and its difficulty distinguishing recent from past (and cured) infections. To evaluate treatment efficacy is still a major concern because direct parasitological methods might overestimate it and the serology has not yet been well evaluated; even if there is a decline in antibody titres after treatment, it is slow and it needs to be done at 6 to 12 months after treatment which can cause a substantial loss to follow-up in a clinical trial.

## Author Summary

Strongyloidiasis is a parasitic infection that can occur in any place of the world. It is not easy to diagnose because the conventional tests are not good enough, especially in individuals that do not present any symptoms of the disease. This is of particular importance in immunocompromised patients, because the disease can spread causing a disseminated disease which can be fatal. In this study, authors review both conventional and novel techniques for the diagnosis of strongyloidiasis. Parasitological examinations based on the detection of the parasite in faeces are the most common techniques used until now in the majority of laboratories. However, they have some disadvantages because most of the best techniques are cumbersome and time consuming and several stool samples have to be collected to improve the diagnosis. New techniques such as the serology which is performed through a blood test are becoming available, but they have still some problems; the test sometimes does not accurately differentiate strongyloidiasis from other helminthic diseases. Another major problem in this disease is to evaluate if patient is cured after the treatment. Parasitological methods can fail to detect treatment failure, and serology has not yet been well evaluated in this context.

## Introduction

*Strongyloides stercoralis* is an intestinal nematode that infects an estimated 30–100 million people worldwide [1]. It is more frequent in areas where hygienic conditions are poor and in areas with a warm and humid climate [2]. Although it generally occurs in subtropical and tropical countries, it might be present in temperate countries with favorable conditions [1]. However, strongyloidiasis can be now diagnosed in non-endemic countries due to the migration flows and travel, being the infection much more common in migrants than in travelers [3].

Risk factors for infection which have identified are HTLV-1 co-infection, malnutrition, chronic obstructive pulmonary disease (COPD), diabetes mellitus (DM), chronic renal failure or breastfeeding [4], [5].

Due to the ability of the parasite to replicate within the host, it is a chronic condition, with a variety of clinical presentations, from asymptomatic patients who are the majority, to hyperinfection with potentially life-threatening dissemination of larvae in immunocompromised patients. They have been summarized in a several reviews [5], [6], [7]

Strongyloidiasis is frequently under diagnosed since many infections remain asymptomatic and conventional diagnostic tests based on parasitological examination are not sufficiently sensitive. Serology is useful but is still only available in reference laboratories. The need for improved diagnostic tests in terms of sensitivity and specificity is clear, particularly in immunocompromised patients or candidates to immunosuppressive treatments. This review aims to evaluate both conventional and novel techniques for the diagnosis of strongyloidiasis. The specific objectives are (i) To review current parasitological tools for the diagnosis of strongyloidiasis, (ii) to review the role of immunodiagnostic tests in strongyloidiasis, (iii) to assess the usefulness of molecular diagnosis of *S.stercoralis* in faecal samples, (iv) to evaluate novel diagnostic tools in the diagnosis of the strongyloidiasis and (v) to review possible cure markers in the follow-up of patients treated for strongyloidiasis.

## Methods

### Search strategy

The search strategy, available at www.cohemi-project.eu, was based on the data-base sources MEDLINE, Cochrane Library Register for systematic review, EmBase, Global Health and LILACS. Other sources of information were also used such as conference proceedings, abstracts, masters and doctoral theses, correspondence with authors from recently published abstracts, and manuscripts in press. Reference lists of all the articles identified were also examined, and relevant cited references were similarly reviewed. The electronic literature search was updated on August 2012. The results were limited in the search string to articles published from 1960 to August 2012 and to English, Spanish, French, Portuguese and German languages. The following search terms were used: “*Strongyloides*” OR “strongyloidiasis” AND “diagnosis”. No restrictions were made with regard to basic study design or data collection (prospective or retrospective). Case reports, case series and animal studies were excluded.

### Study selection

**Figure 1:**
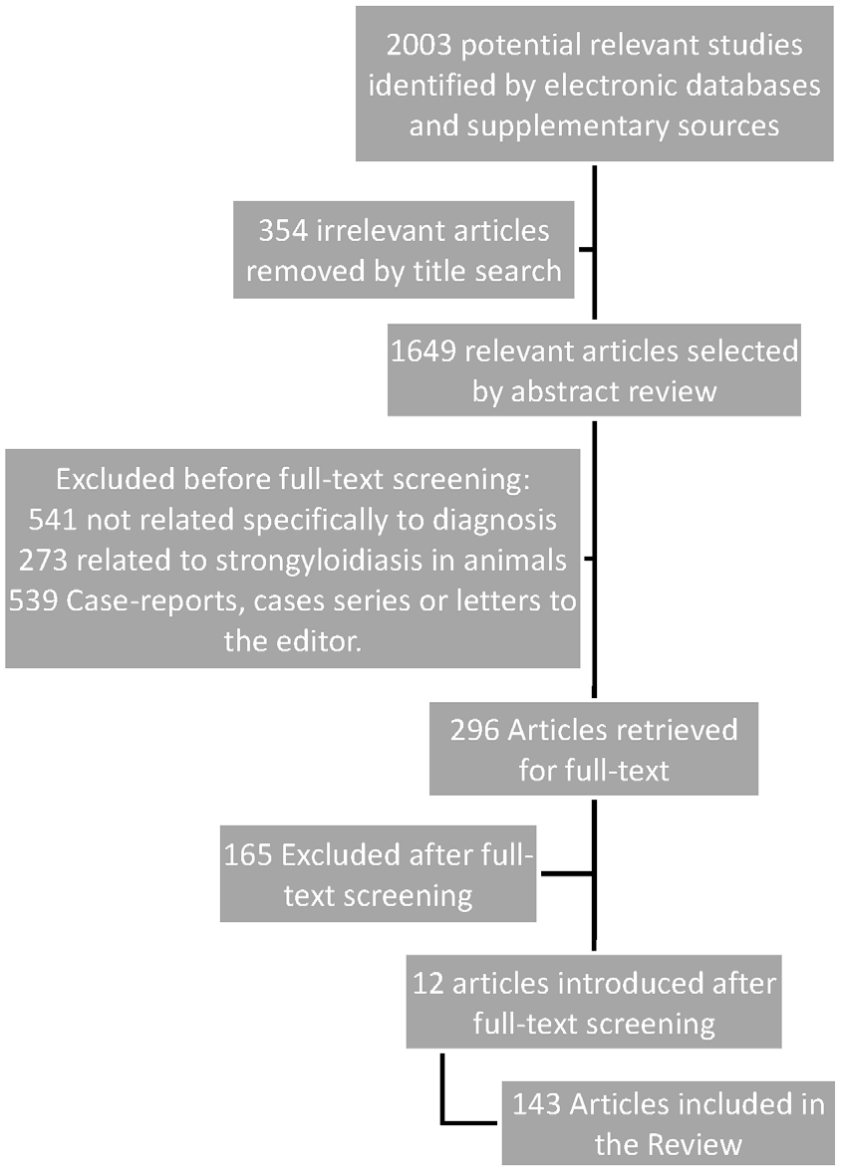
Flow diagram for study selection.

The articles were selected in the following way (see figure 1).

## Results

### Literature search

The study selection process is shown in figure 1. Out of 2003 potentially relevant citations selected for retrieval, 1649 were selected for review of the abstract. Of those, only 296 studies were selected for full-text screening, excluding among them 165 studies and introducing 12 studies whilst reviewing other studies or by other supplementary sources. 143 were eligible for final inclusion.

### Findings

#### Strongyloidiasis and eosinophilia

In returning travelers or migrants, helminthic infection is the commonest identifiable cause of eosinophilia [8]. In many intestinal parasitic infections, eosinophilia can be transient and is associated with the tissue migratory phase of the infection. According to Baaten *et al.*, eosinophilia has a very low positive predictive value (15%) for intestinal parasitic infections in travelers to helminth-endemic countries [9]. Another group found similar results in pediatric refugees (39%), suggesting that eosinophilia might not be of value in screening these populations [10].

However, in *S.stercoralis* infection, eosinophilia might be more frequent compared to other chronic intestinal parasitic infections [11]. A plausible explanation is the fact that the adult female worms live within the submucosa, not in the lumen of the gut and therefore the eosinophilic response might be higher. In this sense, eosinophilia has been considered a potential marker to look further in screening for chronic strongyloidiasis, particularly in asymptomatic individuals [12], [13]. However, eosinophilia in chronic strongyloidiasis might be intermittent and some series of Strongyloidiasis have reported eosinophilia in 57 to 63% of cases [14], [15].

Finally, in patients coming from the tropics with eosinophilia, the precise cause is determined in relatively few cases (15–38%) using conventional methods [16], 17. In a series of former Far East prisoners of the second world War, out of 25 patients with undiagnosed eosinophilia, 11 patients were performed an ELISA antibody test and 45% were diagnosed with strongyloidiasis [12]. Accordingly, in some population groups it is possible that a high proportion of undiagnosed eosinophilia cases might be attributable to underlying *Strongyloides* infection.

The elevation of total serum IgE levels, usually related to eosinophilia has also been reported in *S.stercoralis* infection [18] with rates between 38–59% [19], [20], [21].

#### Faeces examination

Faeces from cases of *S.stercoralis* infection usually contain larvae rather than ova, in contrast to other helminthic parasites. In chronic cases larvae are present only in small numbers, so the number of larvae in the stool may be lower than the detection threshold of the available diagnostic tests. Furthermore, larval excretion may be intermitent which has implications not only for primary diagnosis but also for establishing drug efficacy in clinical trials [22], [23], [24].

Direct faecal smear examination (DS) is a simple and inexpensive method; however a single examination fails to detect 70% of cases compared to a multiple collection of samples. In a study, the maximum detection rate was reached when seven consecutive stool specimen were examined [7], [25]. Some improvements have been proposed to enhance the sensitivity of the DS, such as stimulating the secretion of *S.stercoralis* larvae with albendazole which in a single study led to the detection of *Strongyloides* in 50% of samples that were negative by DS before albendazole intake [26].

There have been many attempts to increase the detection rate of larvae in the faeces and faecal concentration techniques have been widely used for this purpose for a long time.

Formalin-ether concentration technique (FECT) described by Ritchie and later superseded by Allen and Ridley method might improve the sensitivity compared to direct fresh examination although it does not have a high sensitivity either [27], [28].

Some studies have modified the FECT method to try and increase the number of larvae recovered, demonstrating that using fresh stool without a preservative substance and exposing the sample to formalin for a short time (rather than the 10-minutes formalin exposure traditionally used) improved the detection of larvae by 1.8–2.0 times compared to conventional FECT [29]. However, the chemical component used for this technique has been considered hazardous by the US Environmental Protection Agency and many state environmental agencies [30] and it might also not be suitable in limited resource settings. Therefore, some authors have also modified this method introducing other agents such as the formalin Hemo-De concentration procedure [31], or the formalin gasoline procedure [32]. Both seem to be equivalent to FECT though studies with a larger sample size and a broader range of parasites should be performed.

The Baermann method, firstly described in 1917 is a cheap and simple technique based on the ability of *S.stercoralis* to enter a free-living cycle of development [33]. The stool is placed on coarse fabric overlying a mesh screen in a funnel that is filled with warm water and connected to a clamped tube. After an hour's incubation, larvae crawl out of the fecal suspension and migrate into the water, from where they can be collected by centrifugation.

This method has been compared to different conventional methods in several studies, showing that it increases the detection rate by 3.6–4 times compared to the FECT or direct smear [34], [35], [36]. In a study conducted in China comparing DS, an ether concentration technique (ECT), Kato-Katz, Koga agar plate method and Baermann method, the best sensitivity was obtained with the Baermann method (all cases were detected by this method) and both ECT and DS failed to identified even a single case [37]. This technique is labor intensive and it is not usually available in clinical parasitology laboratories but there have been several attempts to reduce the cost and to simplify the technique through slight modifications of the Baermann procedure [38], [39], [40]. Another drawback of the technique is that it requires freshly and non-refrigerated stool samples.

The Harada-Mori technique is a filter-paper culture method which utilizes the water tropism of Strongyloides larvae to concentrate them [41]. Briefly, fresh faeces are deposited on filter paper which is soaked with water and then incubated for 10 days at 30°C. The water sediment is screened daily to look for living larvae [7]. Although it seems to have greater sensitivity compared to FECT or DS [42], the detection rate is inferior compared to the Baermann or agar plate methods, [43], [44], [45], [46]. Furthermore, it is rarely deployed as a standard procedure in clinical parasitology laboratories [7]. Another, simpler approach is the water-emergence method in which a central depression is made in the stool specimen then filled with warm water before incubation for 1 hour at 37°C. During this time larvae crawl out of the faeces and migrate into the water. It has been shown to be more sensitive (85%) compared to DS (56%) or FECT (52%) in a single study [47]. Another relatively simple procedure is the charcoal culture where 1–3 g of faeces are mixed with an equal quantity of coarsely ground charcoal, put on a filter paper which is mounted on a petri dish and incubated for seven days. The sediment of the centrifuged water is examined for the presence of larvae. This technique, described in some large scale surveys in Ghana [48], [49] is particular suitable for the diagnosis of strongyloidiasis in remote regions because it does not require the need of a well equipped laboratory. However, a long incubation period is required and we have not found any study comparing this technique with advanced parasitological methods such as the Baermann method or other cultures.

Early in the 1990s, some groups reported the Agar Plate culture (APC) as a superior method (1.6–6 times more sensitive) compared to traditional methods such DS, filter paper culture or FECT [11], [23], [43], [45], [46], [50], [51], [52], [53], [54], [55]. A study by Sato et al, compared DS, FECT Harada-Mori technique and APC showing that APC was able to detect more than 96% of cases [52]. When APC or Koga agar plate is compared to the Baermann technique, some studies report a better detection rate for the culture [35], [44], [51], [56] though in at least 2 studies, higher sensitivity was obtained by the Baermann method [37], [40].

Briefly, in the APC method, agar culture medium is poured into a sterilized plastic dish equipped with double walls and a solution of glycerin to prevent *S.stercoralis* larvae from getting out of the Petri dish. 3 g of faeces are placed in the agar medium and cultured for 72 hours [24]. Use of fresh faeces is recommended to increase the detection rate.

The larvae leave characteristics tracks on the surface of the agar though microscopy is required to detect the tracks and to differentiate the larvae from those of hookworm [46]. Even if larvae are not found, if these peculiar tracks and bacterial colonies are observed, the diagnosis of Strongyloidiasis should be suspected [24]. It has the disadvantages of being expensive, time consuming and presenting a safety risk for laboratory staff.

In chronic infections, the sensitivity of these methods might not be satisfactory. In the study by Sato et al, the detection rate of APC was still less than 60% if only one sample was tested [52]. Thus, the value of repeated stool examinations to increase the diagnostic yield using the APC or other techniques is widely accepted since it has been demonstrated to result in increased sensitivity [25], [37], [55], [57], [58].

Larvae can also be found in other samples such as sputum, duodenal aspirates, gastric biopsies, cervical smear or CSF liquid, the latter in disseminated *Strongyloides* infections [6], [59], [60], [61], [62], [63], [64], [65].

The string test used for sampling duodenal contents has been shown to be a reliable method for the diagnosis of *S.stercoralis*. Briefly, a nylon yarn coiled inside a lined gelatin capsule is swallowed and the capsule is delivered to the stomach and duodenum. Then the line is pulled back with adhered bile-stained duodenal mucus. Goka et al., compared faecal examination with duodenal fluid obtained by the string test in a group of patients with gastrointestinal symptoms, showing better sensitivity of the latter [66], although other authors have reported lower sensitivity for the string test compared to the direct faecal examination [67]. However, this invasive method should perhaps be recommended only in selected cases eg in an of immunosuppressed patient to maximize the chance of detecting larvae when a prompt diagnosis is essential.

#### Endoscopy

Strongyloidiasis can involve any segment of the gastro intestinal tract. The most common endoscopic appearances, which are often incidental findings, are ulceration, duodenal spasm, bleeding, mucosal edema, thickened duodenal folds, or brown discoloration of the mucosa [62], [68], [69], [70]. Choudry et al., described pustule-like lesions in the colon which may represent the process of larvae burying themselves in the colonic mucosa [62]. Other findings in the colonic mucosa include loss of haustra and narrowing aphtoid ulceration, yellowish-white nodules, erythema or serpiginous ulcerations [69], [71].

**Histological examinations** can confirm the diagnosis showing sections of larvae, eggs and some adult forms, predominantly in the gastric or duodenal crypts with eosinophilic infiltration in the lamina propia, directly correlated with the intensity of infection [71], [72], [73], [74], [75], [76].

#### Diagnostic radiology

There are not pathognomonic radiological findings. A wide variety of signs has been described, ranging from normal appearance of GI tract or mild edema with thickened folds of small bowel mucosa to significant dilatation and a bizarre coarse appearance of the small bowel mucosa with paresis or stricture in hyperinfected patients [77], [78]. Duodenal dilatation or stricture has also been described in barium meal examination s in heavy infections [79]. All these abnormalities are reversible with appropriate therapy [77], [79].

#### Intradermal skin tests

The immediate hypersensitivity reaction in skin to different somatic and excretory/secretory antigens has been reported to be a reliable skin test for the diagnosis of strongyloidiasis though cross-reactions with other nematodes infections have frequently occurred and the persistence of a positive skin test reaction after treatment is also plausible [80], [81]. Moreover, in immunosuppressed patients, particularly those co-infected with human T-Cell Lymphocytotropic virus type 1 infection (HTLV-1,) the immediate hypersensitivity reaction might be reduced leading to a lower sensitivity in this test [82]. Intradermal skin testing is not a realistic option for routine diagnosis of strongyloidiasis.

#### Serology

Several serum antibody detection using a variety of antigens have been already tested over many years. They are summarized in table 1. The particle agglutination test based on indirect immunofluorescence microscopy (IFAT) has been widely used with higher sensitivity than parasitological tests (81–98%) [83], [84], [85], [86], [87]. More recently, Boscolo *et al.* developed an IFAT assay using whole larvae with a high level of diagnostic accuracy at the antibody titer threshold of ≥1∶20 (97% sensitivity and 98% specifity) [87]. The main disadvantage of this technique is its requirement for whole living infective-larvae since the most consistent fluorescence has been found using whole living worm [83], [86]. Therefore, a large amount of larvae is required for its performance. In order to overcome this drawback, Sato *et al.* developed a gelatin particle agglutination (GPAT) test [86] and reported a sensitivity of 81% and a specificity of 74% [88]. Immunoblot analysis has been evaluated against immunodominant antigen of *S.stercoralis* and *S.ratti*, showing sensitivities between 65–100 [89], particularly higher when assessing IgG reactivity to a 41-KD [90] or 26 KDa larval component [91]. Several Enzyme-linked Immunosorbent Assays (ELISAs) have been developed for the diagnosis of strongyloidiasis. There are several in-house ELISAs designed from crude antigenic extracts of filariform larvae, some of them from *S.stercoralis* [92], [93], [94], [95], [96], [97] and others from *S.venezuelensis* or *S.ratti* [98], [99], [100]. Two commercial kits are currently available, the Bordier-ELISA (Bordier Affinity Products) [96] and IVD-ELISA (S-stercoralis serology Microwell ELISA Kit, IVD Research Carlsbad, CA) [96], [101], [102] All of these assays have demonstrated high sensitivity ranging from 73–100%. However, in immunosuppressed patients, the sensitivity of the ELISA is significantly lower, probably due to reduced antibody production [103], [104], [105].

**Table 1:** Characteristics of the main serological tests for strongyloidiasis.

Technique	Sens.	Spec.	Antigen source	Commercial test	Cross -Reactivity	Disadvantages	Reference
IFAT	81–98%	90–98%	*S.stercoralis*	No	XXX	Living infective larvae required. Cross-reactivity with nematode infections.	[83]–[87], [106]
GPAT/IHA	56–81%	74–92%	*S.stercoralis*/*S.ratti*	No	XXX	Cross-reactivity with other nematode infections, particularly with filarial infection in the IHA test. Low sensitivity and specificity, particularly with *S.ratti* crude antigen.	[86], [88], [107]
ELISA - Crude Ag	73–100%	29–93%*	*S.ratti/S.stercoralis/S.venezuelensis/*	BORDIER/IVD research/No/	XXX	Decreased sensitivity in immunosuppressed patients and travelers. Cross-reactivity with other nematode infections. No filarial infections were included to assess cross-reactivity in most of the studies. A large quantity of larvae is required	[92]–[105]
ELISA - Recombinant Ag: NIE	84%	100%	*S.stercoralis*	No	XX	Cross-reactivity with other nematode infections	[119]
ELISA - Preincubation with *O.gutturosa* or other Ag from other nematodes	85–93%	96–97%	*S.stercoralis*	No	X	No cases of filariasis were included to assess cross-reactivity	[90], [108]
WB	65–100%	75–96%	*S.stercoralis* (41 kD, 31 kD, 28 kD) 26 kD/*S.ratti* (11 immunodominant antigens)	No	XX	Cross-reactivity with other nematode infections depending on the immunodominant antigen used. No filarial infections included to assess cross-reactivity.	[89]–[91]
LIPS	97%	100%	*S.stercoralis*	No	No	LIPS is not available in conventional laboratories.	[120]–[121]

Sens: Sensitivity; Spec: Specificity; IFAT: Indirect immunofluorescence test; GPAT: Gelatin particle agglutination test; IHA: Indirect hemagglutination test; ELISA: Enzyme-linked immunoabsorbent assay; WB: Immunoblot test; LIPS: Luciferase immunoprecipitation system.

* Wide variation depending on the study design.

An Iranian research group compared ELISA to IFAT and reported better sensitivity of the ELISA (93.5%) compared to IFAT (87%) [106], [107].Both assays showed false positivity in ascariasis, hydatidosis and toxocariasis although they were less common with the ELISA assay. The considerable cross-reactivity with other nematode species, particularly with filarial infections, is one of the major drawbacks of all these tests (ELISA, GPAT, Immunoblot and IFAT test). This fact is particularly important in evaluating results in migrants, who have been exposed to infection with other helminths over many years, and whose risk of having other nematode infections is higher compared to travelers.

Some authors have proposed a modification of the ELISA consisting of the preincubation of sera with particular nematode extracts, particularly with phosphate-buffered saline-soluble extract of the *Onchocerca gutturosa*, aimed at removing cross-reactivity with other helminths [90], [108]. Conway et al, demonstrated that serum absorption with an extract of *O.gutturosa* could reduce the proportion of false positive results in an indirect ELISA among individuals with filarial or *Necator americanus* infections by more than a half [108].

Several studies have claimed a high sensitivity and specificity for the ELISA test although the study design might have not been adequate. One of these studies compared a panel of sera from patients with known strongyloidiasis with healthy control patients but they did not include sera from individuals harbouring other nematode infections [104]. Other studies have tested the assay on a large serum bank of other parasitic, viral or fungal infections; however, they did not include proven filariasis infections in the assessment of specificity [96], [109].

A case-control study design might overestimate the diagnostic accuracy of the test [110]. Better to assess the performance of the ELISA and other serological tests for the diagnosis of *S.stercoralis*, the ELISA should be tested on populations from endemic countries (population-based studies) where the specificity and consequently the positive predictive value might decrease substantially due to cross-reactivity with other nematode infections. For example, a study conducted by Yori et al, showed that sera from 77% of subjects with known hookworm infections had a positive ELISA result for *S.stercoralis* [*111*].

The difficulty lies in the absence of a reliable gold standard for diagnosis of *S.stercoralis* infection. A study by Gyorkos *et al.* conducted in Canada on newly arrived Asian refugees, showed a sensitivity of 95% and specificity of 29% for the serological test when using stool examination as the gold standard [112]. This was an imperfect gold standard due to its low sensitivity. Hence, we can conclude that serology usually overestimates the burden of disease whereas parasitological techniques underestimate it.

Only in areas where other parasitic diseases are not endemic can the possibility of cross-reactions be reasonably ruled out [113].

On the other hand, a serology test might be less sensitive in returning travelers. Sudarshi *et al.* in a study conducted in London, reported significantly less sensitivity (73%) by ELISA in returning travelers who had been briefly exposed to the parasite compared to migrants in whom the test was 98% sensitive [114]. Larger studies are required further to evaluate this specific issue.

Another problem in assessment of ELISAs for the diagnosis of strongyloidiasis is that the antigens are mostly poorly defined and the laboratory protocols vary substantially [110].

Another disadvantage of using ELISA tests based on crude antigenic extracts from larvae is the large quantity of larvae required to make antigen for use in the test. As is the case with the IFA test, crude antigen-based ELISAs are relatively impractical for a large scale use.

Some proteins on the surface or in the excretory- secretory products of *Strongyloides spp* have been identified in an attempt to improve the serodiagnosis of strongyloidiasis [115], [116], [117]. Moreover, two *S.stercoralis* recombinant antigens 5a and 12 have been characterized and are reported to show no cross-reactivity with sera from patients with filariasis or intestinal nematode infections [118].

Recently, using a recombinant Strongyloides antigen (NIE) developed by Ravi et al. [119], a new test was developed based on the luciferase immunoprecipitation system (LIPS). This assay demonstrated better sensitivity (97%) and specificity (100%) than an NIE-ELISA test. Moreover, there was no cross-reaction with serum from filaria-infected patients. Regarding post-treatment follow-up, a reversion from a positive to a negative result was found more frequently in the NIE-LIPS assays (58%) compared to NIE-ELISA test (17%) although a significantly decline of the antibody response was observed in both assays. [120], [121].

A significant advantage of this test is the use of recombinant antigen which can be purified and produced in large amounts, whereas preparation of crude antigen is often time-consuming and dependent on the collection of faeces from infected humans or experimental animals.

The LIPS technique can be performed rapidly (<2.5 hours), and an even faster version which gives results in less than 2 minutes has been already used for other infections with outstanding results. If applied to *Strongyloides* infection, it could have a valuable role as a rapid diagnostic test. However, this technique is not currently available in the majority of laboratories. Further studies are needed better to explore this method.

#### Coproantigen detection

ELISA has been used to detect coproantigen of *S.stercoralis* in faecal samples from animal models [122], [123], [124] and El-Badry et al, have developed an ELISA able to capture *S.stercoralis* coproantigen from infected patients without cross-reactions with the nematodes (*C.philipinensis*) or with the trematodes (*S.mansoni* and *F.gigantica*) [125] although it was tested on only a very small number of human faecal samples. This could prove to be an easy and inexpensive technique, although more studies are needed on its performance for the diagnosis of strongyloidiasis.

#### Molecular diagnostics

Several real-time PCRs have been designed for the detection of *S. stercoralis*, targeting either 18S rRNA, cythocrome c oxidase subunit I gene, or 28S RNA gene sequences in faecal samples. These tests have generally achieved 100% specificity [126], [127]. Several hyper-variable regions in 18S rDNA of *Strongyloides spp.* have found to be possible markers for species-specific diagnosis [128].When assessed on human samples, the study by Moghaddassani et al, have shown a sensitivity of 100% of a nested PCR performed only in 16 samples compared to APC [129]. Nevertheless, a study performed by Verweij, et al. the test did not achieve better sensitivity than Baermann or APC methods. They pointed out that the amount of fresh faeces used for the assay was 40 times lower than that required for the Baermann or APC method [127]. It must be also borne in mind that the amount of parasite-specific DNA present might be directly correlated with the intensity of the infection and the host immune response [130]. Therefore, in asymptomatic patients with very low levels of larval output, the test is unlikely to achieve high sensitivity unless repeated stool samples are tested.

Some groups have designed multiplex PCR assays to detect as many as 2, 5 or even 7 different intestinal parasites which have resulted in high specificity and with a higher sensitivity than conventional parasitological methods [131], [132], [133], [134].

#### Follow-up

In order to evaluate the results of treatment of strongyloidiasis it is essential to confirm the eradication of *S.stercoralis*, particularly migrating larvae, because of the risk of autoinfection [135]. Since the larval output is intermittent, stool examination might be insufficiently sensitive to evaluate the efficacy of a given treatment [22], [23]. Moreover, positive stool examinations have been described even one or two years after initially negative post treatment results [136]. Accordingly, a negative result should not be interpreted as unequivocal evidence of the absence of infection.

In a study conducted by Dreyer, stool specimens were collected over 8 weeks. Interestingly, 75% of patients who had tested positive for *Strongyloides* infection in one stool examination, had negative results in next four samples although they had not received any treatment. Thus, if these patients had been enrolled in a clinical trial, the efficacy of a placebo drug would have been estimated to be 76% [22]. This has clear implications for the design of clinical trials to determine drug efficacy.

Where parasitological methods are the only techniques available to evaluate drug efficacy, APC or Baermann methods are the most strongly recommended techniques because they have higher sensitivity then DS or FECT. Collection of repeated samples over at least one year is also recommended. However, parasitological methods might not be completely reliable as markers of cure after drug administration.

Antibody detection ELISA could be a suitable alternative, when possible. Some in-house ELISAs have been evaluated as cure markers for strongyloidiasis using reference ranges of positivity determined by the criterion of laboratory concerned [137], [138], [139], [140], [141]. However, a uniform criterion to define cure has not yet been established. The problem is that there is a wide variation in antibody-titres before treatment. Thus, patients with extremely high pre-treatment antibody titres might take longer for the levels to fall below the cut off for positivity treatment and their tests might therefore remain positive at a given time point used for follow-up [138]. This occurs particularly in patients coming from endemic areas or those infected for many years [142]. Several studies have attempted to solve this problem by establishing a cut-off value based on the ratio of post-treatment and pre-treatment optical density (OD) or absorbance observed in the EIA [97], [137], [138]. If the antibody titre decreases remarkably compared to that before treatment, the patient might then be considered to be cured. Kobayashi established a ratio <0.6 (calculated by dividing the follow-up serologic result by the initial result) as an indicator of cure [138]. In the study by Loutfy *et al.*, the antibody levels decreased after treatment and the proportion of patients with a ratio <0.6 increased over time [97]. More research is needed to assess the value of serology as a marker of cure.

Patients should be monitored for 1 or 2 years after the drug therapy to ensure a sustained serological trend suggestive of cure [137]. New ELISA and LIPS assays using the NIE recombinant antigen [121] and also IFAT [87] have been evaluated as cure markers of the disease. Preliminary studies have shown a decreasing trend in antibody-titre after treatment and a significant difference between antibody titres pre and post-treatment [143] though a reliable cut-off value has not yet been established.

## Discussion

Strongyloidiasis is a neglected parasitic disease the prevalence of which might be underestimated in many countries and has a particularly importance in immunosuppressed patients because of the risk of hyperinfection. It does not have characteristic clinical features apart from larva currens, although eosinophilia is usually common among infected patients. Stool examination is still considered the primary technique for the detection of *S.stercoralis* infection.

Since direct microscopic examination was first used, many other parasitological methods have been implemented, improving considerably the detection rate of *S.stercoralis* larvae in faeces. Several specimens should be collected on different days to improve detection rate. However, the sensitivity of microscopic-based techniques might not be good enough, especially in chronic infections where larval output is very low. Furthermore, techniques such as Baermann or APC are cumbersome, time-consuming and are not currently deployed in most laboratories.

Serology remains a useful tool both only for epidemiological studies and for the diagnosis of individual cases. However, it might overestimate the prevalence of disease due to cross-reactivity with other nematode infections. Recently, the use of a recombinant antigen (NIE) applied to the LIPS technique instead of ELISA has shown a promising reduction of cross-reactivity although more studies are required to confirm this. Another major problem in strongyloidiasis is to evaluate treatment efficacy, since direct parasitological methods might overestimate it and serology has not yet been well evaluated in this context. Although some studies have shown a clear tendency to decline in antibody titer after treatment, a clear cut-off value needs still to be defined. The slow decline means that serological testing needs to be done at 6 to 12 months after treatment which can cause a substantial loss to follow-up in a clinical trial.

It is not yet clear as to the dose of ivermectin to eradicate *Strongyloides* infection, so further efficacy trials must be conducted. The lack of a reliable method to evaluate cure is a major concern in a trial design. Therefore, identification and evaluation of a valid cure marker should be undertaken before conducting these trials.

In summary, there is an urgent need of new tools to diagnose strongyloidiasis: efforts are being done to improve specificity of current and new serological methods and their value as a reliable cure marker. Another option is to combine different diagnostic methods as a composite diagnosis to improve sensitivity and specificity for clinical trials, and situations requiring high diagnostic accuracy. In parallel, the development of new biomarkers to evaluate cure of the disease is urgently needed.

This research need has been identified by COHEMI network as one of the major gaps in the management of strongyloidiasis.

## Supporting Information

Checklist S1 **PRISMA checklist.**
(DOC)

Flowchart S1 **PRISMA flowchart.**
(DOC)
