# Supplementary material for: The Laboratory Diagnosis and Follow Up of Strongyloidiasis: A Systematic Review
Source: PLoS Negl Trop Dis. 2013 Jan 17;7(1):e2002. doi: 10.1371/journal.pntd.0002002 (PMC3547839; doi:10.1371/journal.pntd.0002002)
Supplement: Flowchart S1 — PRISMA flowchart. (DOC) [file pntd.0002002.s002.doc]

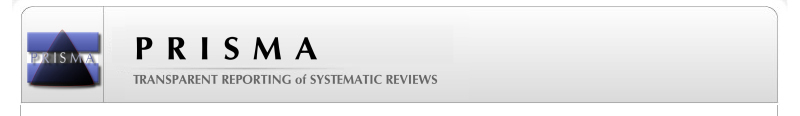
**PRISMA 2009 Flow Diagram**

**Screening**

**Included**

**Eligibility**

**Identification**

Records identified through database searching
(n =3202 )

Additional records identified through other sources
(n = 12)

Records after duplicates removed
(n =2003)

Records screened
(n =1649)

Records excluded
(n =1353)

Full-text articles assessed for eligibility
(n = 296)

Full-text articles excluded, with reasons
(n = 165)

Studies included in qualitative synthesis
(n =143)

Studies included in quantitative synthesis (Serology)
(n =31)
